# Metal-insulator-semiconductor (MIS) photoelectrodes: distance improves performance

**DOI:** 10.1093/nsr/nwab089

**Published:** 2021-05-24

**Authors:** Joshua Jack, Zhiyong Jason Ren

**Affiliations:** Department of Civil and Environmental Engineering and the Andlinger Center for Energy and the Environment, Princeton University, USA; Department of Civil and Environmental Engineering and the Andlinger Center for Energy and the Environment, Princeton University, USA

Hydrogen (H_2_) is a clean energy carrier with enormous potential to support deep decarbonization towards net-zero and a circular carbon economy. However, H_2_ production itself can be carbon and energy intensive, as 96% of global H_2_ synthesis is currently conducted via thermochemical processes such as steam reforming and coal gasification that rely on fossil fuels [1].

Photoelectrochemical (PEC) water-splitting is a promising method to decarbonize H_2_ production by directly converting low cost and abundant solar energy into H_2_ with little or no external bias [2]. Crystalline Si has been shown to be an excellent photoelectrode material and is capable of supporting both the oxygen evolution reaction (OER) at the anode and hydrogen evolution reaction (HER) at the cathode. Contemporary studies have favored the use of metal-insulator-semiconductor (MIS) photoelectrodes over traditional p–n junction designs because of their simple fabrication and potential to achieve higher efficiencies [3]. MIS junction electrodes have conventionally used p-Si photocathodes where catalysts and metals are deposited on the same side as light illumination, leading to large amounts of parasitic light absorption that can lower current densities and overall efficiencies [3].

In a recent report by Jinlong Gong and co-workers at Tianjin University, a new n-Si based MIS electrode was developed to spatially decouple light absorption from reaction sites, which effectively mitigated the long-standing hurdle of detrimental light capture by catalysts/metals on p-Si photocathodes [4].

**Figure 1.**
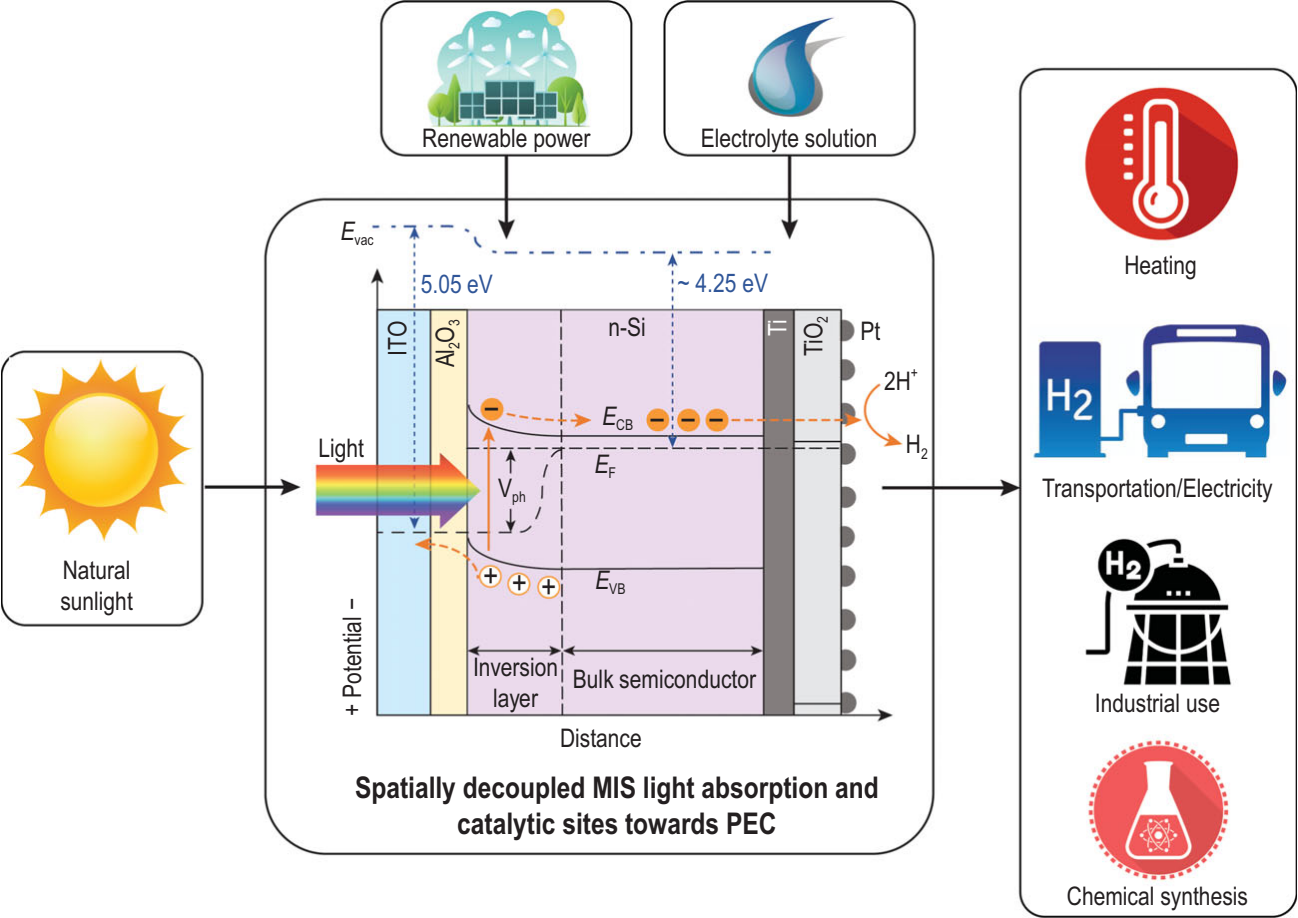
Hydrogen production and utilization process using spatially decoupled light absorption and catalytic sites in metal-insulator-semiconductor (MIS) photoelectrodes [4].

In their study, the researchers utilized an n-Si semiconductor that has a lower Fermi-level than p-Si, enabling the use of a wide range of high work function metals that create large band offsets. Figure 1 shows a schematic of their proposed electrode design where light travels through the n-Si/Al_2_O_3_/ITO MIS junction and water is reduced to H_2_ at the Pt catalyst on the backside. Importantly, metallic indium tin oxide (ITO) is the only material that determines the parasitic light absorption, and its high light transmittance enables the adoption of a thick layer that can generate a large photovoltage [4].

To highlight this new feature, the authors quantified the anti-reflective nature of the ITO film and compared the amount of light absorbed in this n-Si photocathode to that in conventional p-Si photocathodes using Ti layers. Under illumination, the proposed n-Si photoelectrode demonstrated excellent PEC performance with better current densities, onset potential and applied bias photo-to-current efficiency than many traditional p-Si based MIS photocathodes. Similarly, high current densities and solar-to-hydrogen efficiencies were achieved in an unassisted tandem cell when pairing the n-Si MIS photocathode with a BiVO_4_/FeOOH/NiOOH photoanode.

The exciting advancements described by Gong *et al.* hold the potential to eliminate the trade-off between beneficial metal/catalyst coverage and decreased light absorption and are applicable to a broad range of photocatalytic reactions. In fact, the researchers also demonstrated the versatility of the n-Si electrode as a photoanode and achieved higher current densities and stabilities than comparable photoelectrodes used for the OER.

Previous designs involving transparent nanoscale-structured metals/catalysts and 3-D reactor architectures, that minimize catalyst geometric footprints, have also sought to eliminate the light absorption problem in MIS junction electrodes [5]. As such, it is feasible that future designs can further boost PEC performance by combining several of these techniques with various high work function metals made available by n-Si based electrodes and the novel MIS junction designs.

***Conflict of interest statement.*** None declared.
